# Association between metabolic body composition status and risk for impaired renal function: A cross-sectional study

**DOI:** 10.1371/journal.pone.0223664

**Published:** 2019-11-26

**Authors:** Wen-Cheng Li, Jau-Yuan Chen, Yu-Ping Liu, Yi-Yen Lee, Wei-Chung Yeh, Wei Yu, Yu-Chung Tsao

**Affiliations:** 1 Department of Family Medicine, Chang Gung Memorial Hospital, Linkou, Taoyuan, Taiwan; 2 College of Medicine, Chang Gung University, Taoyuan, Taiwan; 3 Department of Health Management, Xiamen Chang-Gung Hospital, Xiamen, China; 4 Department of Endocrinology and Metabolism, Xiamen Chang-Gung Hospital, Xiamen, China; 5 Division of Pediatric Neurosurgery, Neurological Institute, Taipei Veterans General Hospital, Taipei, Taiwan; 6 Faculty of Medicine, National Yang-Ming University, Taipei, Taiwan; 7 Department of Occupational Medicine, Chang Gung Memorial Hospital, Linkou, Taoyuan, Taiwan; National Yang-Ming University, TAIWAN

## Abstract

**Background:**

The risk for obesity-related disorders is proportional to the visceral region and had been observed to be highly related with impaired renal function. In the current study, we aimed to evaluate renal function impairment, according to sex, age, and different status of metabolic body composition.

**Methods:**

We retrospectively collected from the medical records the basic information and metabolic titers of Chinese adults (13,373 men and 10,175 women) who underwent health checkup from 2013 to 2016. The population was divided into four groups, according to metabolic body composition, including metabolic healthy norms-weight (MHNW), metabolic healthy obesity (MHO), metabolic unhealthy norms-weight (MUNW), and metabolic unhealthy obesity (MUO). The categorical data were compared among the groups and logistic regression analyses were conducted to investigate the association between metabolic body composition status and risk for renal function impairment.

**Results:**

Across all ages in both sexes, the odds ratios (OR) for renal function impairment were higher in the MHO, MUNW, and MUO groups than in the MHNW group, except for women <45 years old in the MUNW group. However, after adjustment, the trend was no longer significant in all groups under 45 years old. For individuals >45 years old, the relatively high risk for renal function impairment remained significantly associated with the MUNW group (OR 2.95, 95% CI 2.02–4.30 in men and OR 1.95, 95% CI 1.35–2.82 in women) and MUO group (OR 2.33, 95% CI 1.82–3.00 in men and OR 2.67, 95% CI 2.04–3.48 in women).

**Conclusion:**

Impaired renal function was independently associated with the status of metabolic obesity. However, the trend was only observed in individuals >45 years old, with significant sex difference.

## Introduction

The risk for obesity-related disorders is proportional to the extent of obesity [1, 2], especially the accumulation of visceral fat [3]. However, some obese individuals may not have an increased risk for the development of metabolic abnormalities; this clinical condition had been referred to as metabolically healthy obesity (MHO) [2, 4]. Although classified as obese, these people do not show insulin resistance or metabolic syndrome in their blood tests. Body mass index (BMI) had been the widely used indicator of obesity, but it may have limitations in the assessment of metabolic or health conditions, particularly in individuals classified as obese [5]; this seemed to be accounted for by the lower level of subcutaneous fat and lipid accumulation in the liver in some types of obesity than in the other types, despite similar body fat rates.

The peculiarity in MHO patients had been explained by several mechanisms, including the significantly low levels of harmful metabolic processes, such as inflammation [6]; higher lipolysis [7]; increased physical activity; low levels of uric acid [8]; and a less degree of liver enzyme concentration due to a low liver fat [9]. Moreover, the insulin sensitivity, blood pressure, blood lipids, and inflammatory markers [i.e., plasma C-reactive protein (CRP)] of MHO individuals were reported to be normal [10, 11]. In addition, in an 11-year study, the risks for CVD and diabetes were similar between normal-weight subjects and MHO individuals [12]. On the other hand, the metabolism of metabolic unhealthy norms-weight (MUNW) individuals (i.e., normal BMI) is similar with the metabolism typical obese individuals, who present with impaired insulin sensitivity; increased visceral obesity; low levels of high-density lipoprotein cholesterol (HDL-C), fasting blood sugar, and triglycerides (TG); and high blood pressure levels [13–19]. Compared with control subjects, MUNW patients had been associated with three to four times higher risk for type 2 diabetes. However, compared with patients with obesity insulin resistance, MHO patients had been associated with three to four times lower risk for type 2 diabetes [12].

The risk factors of cardiovascular and metabolic diseases had been related to inflammation of the blood vessels, particularly the small blood vessels, which can lead to chronic kidney disease (CKD). In fact, some studies showed the effects on the small blood vessels in the kidneys [20]. Microalbuminuria is an early indicator of diabetic nephropathy and occurs 5 to 10 years before the onset of apparent proteinuria, which is a sign of a more progressive kidney disease and had been a previously used marker of renal failure. Mogensen was the first to describe microalbuminuria as a strong predictor of renal dysfunction, as well as cardiovascular disease mortality in patients with diabetes[21]. In recent years, microalbuminuria had been receiving increasing attention as a prognostic marker for cardiovascular and/ or renal dysfunction in non-diabetic individuals [22].

After our thorough literature search, the association of renal function impairment with sex, age, and metabolic obesity had been rarely reported. We assumed that MHO, MUNW, and metabolic unhealthy obese (MUO) individuals differ in the correlation of albuminuria and eGFR with the cardiac metabolism of inflammatory markers. We aimed to compare these individuals, in terms of the impact of sex, age, and ethnic group on the risk for kidney damage.

## Materials and methods

We retrospectively collected the medical examination records of Chinese adults (aged ≥18 years) who underwent health checkups from 2013 to 2016 at Chang Gung Memorial Hospital. Subjects with incomplete data; history of any chronic disease, such as thyroid or hypothalamic disease, adrenal gland disease, renal cancer, postrenal transplantation, glomerulonephritis, nephritic syndrome, hepatocellular carcinoma, and cirrhosis; intake of medications that may affect the metabolic status or kidney function (e.g. diuretics, thyroid medications, or renal replacement therapy); and pregnant women were excluded from this study. A total of 13,373 men and 10,175 women were included for analysis. The study was approved by the institutional review board of Chang Gung Memorial Hospital (XMCGIRB2018005) and was conducted in accordance with the guidelines of the Helsinki Declaration. Inform consent was not obtained due to the setting of retrospective record analyzing with all the data accessed anonymously.

At the beginning of the health examination, trained nurses collected the data, using a standardized questionnaire that comprised information on history of past illnesses, medications, and physiologic conditions. This was followed by a detailed physical examination, including measurements of weight (kg), height (cm), waist circumference (cm), and blood pressure (mmHg). BMI was calculated as the body weight divided by the square of the height (kg/m^2^). Body height and weight were measured using calibrated meters and scales, according to a standard protocol. Wait circumference was measured midway between the lowest rib and the iliac crest. Blood pressure was measured three times, using an automated sphygmomanometer, after placing the participant in a seated position for at least 15 minutes. Up to three measurements were averaged for systolic blood pressure (SBP) and diastolic blood pressure (DBP). The mean arterial pressure (MAP) was estimated as (2/3) × DBP + (1/3) × SBP.

The laboratory data included fasting blood samples for total cholesterol (TCHOL); low-density lipoprotein cholesterol (LDL-C, mmol/L); HDL-C (mmol/L); TG (mmol/L); fasting blood glucose (FBG, mmol/L); serum creatinine (SCr); high sensitivity CRP (hsCRP, μg/mL); and insulin, which were determined by enzymatic, spectrophotometric, or colorimetric methods. Urine was collected for microalbuminuria and creatinine. All data were entered into a centralized electronic database, under strict quality control, with monitoring at a regular basis.

Based on the CKD-EPI creatinine equation from the Chronic Kidney Disease Epidemiology Collaboration (CKD-EPI) [23], SCr (1 mg/dL = 88.4 μmol/L) was used to the estimate glomerular filtration rate (eGFR), which was calculated as

eGFR(mL/min/1.73m2)=141×min(SCrκ,1)α×max(SCrκ,1)−1.209×0.993Age×1.018[iffemale]×1.159[ifBlack]

κ = 0.7 (females) or 0.9 (males); α = -0.329 (females) or -0.411 (males); min = indicates the minimum of S_Cr_/κ or 1; max = indicates the maximum of S_Cr_/κ or 1; age = years

On the basis of the recommended ACR cutoff values, the urinary secretion of albumin was classified as normoalbuminuria (ACR < 30 mg/g Cr); microalbuminuria (ACR 30–299 mg/g Cr); or macroalbuminuria (ACR > 300 mg/g Cr) [24]. Based on the definition of the Kidney Disease Outcomes Quality Initiative [25], the participants were classified as CKD stage 1 (eGFR ≥ 90 mL/min/1.73 m^2^ with proteinuria); CKD stage 2 (eGFR 60–89 mL/min/1.73 m^2^ with proteinuria); CKD stage 3 (eGFR 30–59 mL/min/1.73 m^2^); CKD stage 4 (eGFR 15–29 mL/min/1.73 m^2^); and CKD stage 5 (eGFR < 15 mL/min /1.73 m^2^). The definition of renal function impairment in the current study is positive albuminuria (ACR ≥ 30 mg/g Cr) or normoalbuminuria with eGFR < 60 mL/min/1.73 m^2^.

A diagnosis of metabolic syndrome (MS) was made in a subject who presented with at least three of the five factors described by the Third Adult Treatment Panel of the National Cholesterol Education Program. The five factors are high blood pressure (a systolic blood pressure ≥ 130 mmHg and/or diastolic pressure ≥ 85 mmHg, under treatment, or already diagnosed with hypertension); high serum triglyceride (≥1.7mmol/ L or under treatment); decreased HDL-C (<1.03mmol/ L for males and < 1.29mmol/ L for females or under treatment); hyperglycemia (FBG ≥5.6mmol/ L, under treatment, or previously diagnosed with diabetes mellitus); and abdominal obesity defined by waist circumference (waist circumference ≥ 90 cm for men and ≥ 80 cm for women). Homeostasis model assessment-insulin resistance (HOMA-IR) was used to quantify insulin resistance, according to the following formula:

fastinginsulin(mIU/L)×FBG(mmol/L)22.5

The participants were divided into four groups of metabolic body composition, including metabolically metabolic healthy norms-weight (MHNW) (HOMA-IR < 2.5 without MS, BMI < 25); metabolic healthy obesity (MHO) (HOMA-IR < 2.5 without MS, BMI ≥ 25); metabolic unhealthy norms-weight(MUNW) (HOMA-IR ≥ 2.5 or with MS, BMI < 25); and metabolic unhealthy obesity(MUO) (HOMA-IR ≥ 2.5 or with MS, BMI ≥ 25). The cut-off value HOMA-IR as an indicator of metabolic syndrome was based on two recent studies of Asian population [26, 27].

### Statistical analysis

The continuous data were compared according to sex, groups of metabolic body composition, and BMI, using t-test or one-way analysis of variance. The categorical data among the groups were compared using chi-square test. Bonferroni *posthoc* comparison was performed for pairwise comparisons among the study groups.

To investigate the association between metabolic body composition and the risk for renal function impairment, we conducted logistic regression analyses without and with covariates adjustment. We chose mean arterial pressure, total cholesterol, triglycerides / HDL cholesterol, and hs-CRP level as the covariates. HOMA-IR, sex, and age were grouping variables, thus they were not adjusted. Metabolic body composition was variable of interest, therefore body mass index and waist-to-height ratio were not adjusted. Fasting blood glucose and insulin are highly correlated to HOMA-IR, so they were also not adjusted. LDL cholesterol was not adjusted due to its collinearity with total cholesterol. Both Triglycerides and HDL cholesterol were not adjusted because of their collinearity with triglycerides / HDL cholesterol. *P < 0*.05 was considered statistically significant. Data analyses were conducted using SPSS 22 (IBM SPSS, IBM Corp, Armonk, NY).

## Results

### Characteristics of the study subjects

Details of the main characteristics of the study subjects, according to sex, are shown in Table 1. A total of 23,548 patients were enrolled in this study. Overall, the median age was 46 years with a range from 18 to 93 years (data not shown). The mean age of the participants was 47.0 years (SD 10.4 years) for men and 47.6 years (SD 10.7 years) for women. The BMI and WHR were 24.6 kg/m^2^ and 0.514, respectively, for men and 23.0 kg/m^2^ and 0.500, respectively, for women. The MAP, FBS, TCHOL, TG, LDL-C, TG/HDL-C, hsCRP, insulin, and HOMA-IR levels were significantly higher in men than in women (all *P < 0*.001). On the other hand, HDL-C, eGFR, and ACR were lower in men than in women. In addition, the proportions of MHO and MUO were higher in men than in women, but the proportion of MHNW was higher in women than in men. The prevalence of impaired renal function was not significantly different between sexes (5.5% for men vs. 6.0% for women, *P* = 0.098).

**Table 1:** Main characteristics of the study subjects by sex.

Characteristics	Men	Women	*P* value
Number	13373	10175	−
Age (years)	47.0 (10.4)	47.6 (10.7)	<0.001
Body mass index (kg/m^2^)	24.6 (3.2)	23.0 (3.2)	<0.001
Waist-to-height ratio	0.514 (0.051)	0.500 (0.060)	<0.001
Systolic blood pressure (mmHg)	121.6 (16.9)	115.9 (19.9)	<0.001
Diastolic blood pressure (mmHg)	76.3 (11.4)	68.8 (11.1)	<0.001
Mean arterial pressure (mmHg)	91.4 (12.6)	84.5 (13.3)	<0.001
Fasting blood glucose (mmol/L)	5.48 (1.48)	5.25 (1.08)	<0.001
Total cholesterol (mmol/L)	5.26 (0.97)	5.13 (0.97)	<0.001
Triglycerides (mmol/L)	1.78 (1.41)	1.15 (0.84)	<0.001
LDL cholesterol (mmol/L)	3.43 (0.87)	3.19 (0.86)	<0.001
HDL cholesterol (mmol/L)	1.19 (0.28)	1.43 (0.31)	<0.001
Triglycerides/ HDL-C	1.78 (1.41)	1.15 (0.84)	<0.001
hsCRP (μg/mL)	2.16 (4.88)	1.56 (3.48)	<0.001
Insulin (mIU/L)	6.94 (3.93)	6.50 (3.25)	<0.001
HOMA-IR	1.71 (1.15)	1.54 (0.94)	<0.001
Study group, n (%)			<0.001
MHNW	7,117 (53.2)	7,117 (69.9)	
MHO	4,020 (30.1)	1,869 (18.4)	
MUNW	452 (3.4)	464 (4.6)	
MUO	1,784 (13.3)	725 (7.1)	
eGFR (mL/min/1.73 m^2^)	97.0 (13.2)	102.6 (13.1)	<0.001
ACR (mg/g Cr)	10.0 (36.1)	12.9 (43.9)	<0.001
Renal function impairment, n (%)	738 (5.5)	613 (6.0)	0.098

hsCRP, high sensitivity C-reactive protein; HOMA-IR, homeostasis model assessment-insulin resistance; MHNW, metabolic healthy norms-weight; MHO, metabolic healthy obesity; MUNW, metabolic unhealthy norms-weight; MUO, metabolic unhealthy obesity; ACR, albumin–creatinine ratio; Cr, creatinine

Data are presented as mean (*SD*) or percentage (%)

### Characteristics of men, according to metabolic body composition status and BMI

The baseline characteristics of men, according to metabolic body composition status and BMI and stratified by age are presented in Table 2. Subjects were classified into four groups: MHNW (3131 of <45 years old and 3986 of ≥45 years old), MHO (1917 of <45 years old and 2103 of ≥45 years old), MUNW (180 of <45 years old and 272 of ≥45 years old), and MUO (876 of <45 years old and 908 of ≥45 years old).

**Table 2:** Baseline characteristics of the male subjects, according to metabolic body composition status and BMI, stratified by age.

Characteristics	MHNW	MHO	MUNW	MUO	*P* value
<45 years old					
Number	3131	1917	180	876	−
Age (years)	37.9 (5.0)	38.9 (4.4)^a^	39.1 (4.3)^a^	38.0 (4.9)^bc^	<0.001
Body mass index (kg/m^2^)	22.2 (2.0)	27.2 (1.8)^a^	23.6 (1.3)^ab^	28.8 (2.7)^abc^	<0.001
Waist-to-height ratio	0.474 (0.037)	0.542 (0.034)^a^	0.503 (0.024)^ab^	0.564 (0.041)^abc^	<0.001
Mean arterial pressure (mmHg)	86.8 (10.6)	93.2 (12.1)^a^	92.4 (11.8)^a^	97.7 (12.6)^abc^	<0.001
Fasting blood glucose (mmol/L)	5.07 (0.98)	5.16 (0.66)	6.41 (2.82)^ab^	6.11 (2.07)^abc^	<0.001
Total cholesterol (mmol/L)	5.10 (0.91)	5.29 (0.96)^a^	5.36 (1.00)^a^	5.51 (0.99)^ab^	<0.001
Triglycerides (mmol/L)	1.47 (1.09)	2.01 (1.47)^a^	2.23 (1.63)^a^	2.76 (2.02)^abc^	<0.001
LDL cholesterol (mmol/L)	3.30 (0.82)	3.49 (0.89)^a^	3.51 (0.90)^a^	3.56 (0.93)^a^	<0.001
HDL cholesterol (mmol/L)	1.27 (0.29)	1.12 (0.23)^a^	1.15 (0.28)^a^	1.06 (0.20)^abc^	<0.001
Triglycerides/ HDL-C	1.29 (1.26)	1.93 (1.76)^a^	2.12 (1.65)^a^	2.78 (2.42)^abc^	<0.001
hsCRP (μg/mL)	1.49 (3.54)	2.16 (3.83)^a^	2.16 (4.41)	3.23 (6.50)^abc^	<0.001
Insulin (mIU/L)	5.34 (2.07)	7.24 (2.09)^a^	12.37 (3.31)^ab^	14.38 (4.81)^abc^	<0.001
HOMA-IR	1.20 (0.51)	1.63 (0.49)^a^	3.29 (0.93)^ab^	3.75 (1.33)^abc^	<0.001
eGFR (mL/min/1.73 m^2^)	103.9 (11.3)	101.4 (11.8)^a^	104.2 (10.7)^b^	103.9 (12.2)^b^	<0.001
ACR (mg/g Cr)	6.3 (32.4)	7.5 (22.4)	12.1 (45.4)	14.8 (41.7)^ab^	<0.001
Renal function impairment, n (%)	68 (2.2)	65 (3.4)	10 (5.6)^a^	81 (9.2)^abc^	<0.001
≥45 years old					
Number	3986	2103	272	908	−
Age (years)	55.1 (7.9)	53.6 (7.5)^a^	53.3 (6.6)^a^	53.7 (7.6)^a^	<0.001
Body mass index (kg/m^2^)	22.3 (1.9)	26.9 (1.6)^a^	23.6 (1.2)^ab^	28.0 (2.3)^abc^	<0.001
Waist-to-height ratio	0.489 (0.038)	0.552 (0.033)^a^	0.517 (0.028)^ab^	0.570 (0.041)^abc^	<0.001
Mean arterial pressure (mmHg)	89.1 (12.2)	95.0 (12.5)^a^	93.3 (11.8)^a^	98.9 (13.2)^abc^	<0.001
Fasting blood glucose (mmol/L)	5.33 (1.16)	5.41 (0.90)	7.97 (3.54)^ab^	6.82 (2.37)^abc^	<0.001
Total cholesterol (mmol/L)	5.23 (0.97)	5.32 (0.97)^a^	5.43 (1.01)^a^	5.39 (1.06)^a^	<0.001
Triglycerides (mmol/L)	1.44 (1.07)	1.87 (1.36)^a^	2.19 (1.69)^ab^	2.45 (1.89)^abc^	<0.001
LDL cholesterol (mmol/L)	3.40 (0.87)	3.51 (0.86)^a^	3.54 (0.91)	3.48 (0.94)	<0.001
HDL cholesterol (mmol/L)	1.26 (0.30)	1.13 (0.23)^a^	1.12 (0.27)^a^	1.07 (0.23)^ab^	<0.001
Triglycerides/ HDL-C	1.27 (1.26)	1.81 (1.91)^a^	2.16 (1.99)^ab^	2.48 (2.27)^abc^	<0.001
hsCRP (μg/mL)	2.15 (5.76)	2.25 (4.10)	2.95 (6.57)	2.98 (5.55)^ab^	<0.001
Insulin (mIU/L)	4.76 (1.98)	6.57 (2.08)^a^	10.93 (4.00)^ab^	12.78 (4.32)^abc^	<0.001
HOMA-IR	1.12 (0.50)	1.55 (0.51)^a^	3.50 (1.28)^ab^	3.72 (1.47)^abc^	<0.001
eGFR (mL/min/1.73 m^2^)	92.1 (11.7)	91.0 (12.6)^a^	92.9 (13.3)	92.0 (13.2)	0.002
ACR (mg/g Cr)	8.7 (31.1)	11.4 (34.5)	25.2 (93.8)^ab^	20.9 (50.1)^ab^	<0.001
Renal function impairment, n (%)	183 (4.6)	150 (7.1)^a^	40 (14.7)^ab^	141 (15.5)^ab^	<0.001

hsCRP, high sensitivity C-reactive protein; HOMA-IR, homeostasis model assessment-insulin resistance; MHNW, metabolic healthy norms-weight; MHO, metabolic healthy obesity; MUNW, metabolic unhealthy norms-weight; MUO, metabolic unhealthy obesity; ACR, albumin-creatinine ratio; Cr, creatinine

^a,b,c^significant *posthoc* comparisons vs. MHNW, MHO, and MUNW, respectively

Data are presented as mean (*SD*) or percentage (%)

Among men under 45 years old, there were no significant differences in the metabolic biomarkers (i.e., TCHOL, TG, LDL-C, and HDL-C); ACR; and prevalence of renal function impairment between the MHO and MUNW groups. In contrast, the MUO and MUNW differed in two metabolic biomarkers (i.e., TG and HDL-C) and prevalence of renal function impairment but not in the ACR.

Among men over 45 years old, the results were quite similar to those in the younger population. Compared with the MHO group, the MUNW group had higher metabolic biomarkers (i.e., TG and TG/HDL-C); ACR; and prevalence of renal function impairment. Notably, although the MUO group demonstrated higher metabolic biomarkers (i.e., TG, TG/HDL-C), compared with those in the MUNW group, there were no significant group differences in eGFR, ACR, and prevalence of renal function impairment.

### Characteristics of women, according to metabolic body composition status and BMI

Table 3 shows the baseline characteristics of women, according to metabolic body composition status and BMI, stratified by age. Subjects were classified into four groups: Healthy (3541 of <45 years old and 3576 of ≥45 years old), MHO (369 of <45 years old and 1500 of ≥45 years old), MUNW (150 of <45 years old and 314 of ≥45 years old), and MUO (153 of <45 years old and 572 of ≥45 years old).

**Table 3:** Baseline characteristics of the women, according to metabolic body composition status and BMI, stratified by age.

Characteristics	MHNW	MHO	MUNW	MUO	*P* value
<45 years old					
Number	3541	369	150	153	−
Age (years)	37.2 (5.2)	39.1 (4.2)^a^	37.2 (4.9)^b^	39.1 (4.3)^ac^	<0.001
Body mass index (kg/m^2^)	20.9 (2.0)	26.7 (1.9)^a^	22.9 (1.6)^ab^	28.1 (2.5)^abc^	<0.001
Waist-to-height ratio	0.457 (0.041)	0.534 (0.038)^a^	0.490 (0.038)^ab^	0.558 (0.047)^abc^	<0.001
Mean arterial pressure (mmHg)	77.4 (9.2)	83.4 (11.8)^a^	82.1 (11.5)^a^	89.2 (13.4)^abc^	<0.001
Fasting blood glucose (mmol/L)	4.92 (0.48)	5.06 (0.46)^a^	5.48 (0.81)^ab^	5.80 (1.45)^abc^	<0.001
Total cholesterol (mmol/L)	4.74 (0.81)	5.00 (1.00)^a^	4.88 (0.84)	4.91 (0.78)	<0.001
Triglycerides (mmol/L)	0.83 (0.44)	1.14 (0.68)^a^	1.34 (0.96)^ab^	1.63 (1.06)^abc^	<0.001
LDL cholesterol (mmol/L)	2.84 (0.72)	3.22 (0.88)^a^	3.12 (0.77)^a^	3.18 (0.69)^a^	<0.001
HDL cholesterol (mmol/L)	1.50 (0.31)	1.33 (0.27)^a^	1.27 (0.26)^a^	1.20 (0.21)^ab^	<0.001
Triglycerides/ HDL-C	0.60 (0.44)	0.92 (0.69)^a^	1.18 (1.30)^ab^	1.47 (1.16)^abc^	<0.001
hsCRP (μg/mL)	0.95 (3.03)	1.63 (2.55)^a^	1.52 (1.95)	2.97 (3.26)^abc^	<0.001
Insulin (mIU/L)	5.46 (2.03)	7.20 (2.07)^a^	12.78 (2.90)^ab^	13.66 (2.90)^abc^	<0.001
HOMA-IR	1.19 (0.48)	1.60 (0.50)^a^	3.08 (0.70)^ab^	3.48 (0.97)^abc^	<0.001
eGFR (mL/min/1.73 m^2^)	111.9 (9.8)	109.1 (9.8)^a^	113.0 (11.1)^b^	110.6 (8.4)	<0.001
ACR (mg/g Cr)	7.7 (16.9)	12.0 (45.3)^a^	12.2 (48.8)	14.6 (29.6)^a^	<0.001
Renal function impairment, n (%)	80 (2.3)	17 (4.6)^a^	5 (3.3)	10 (6.5)^ac^	0.001
≥45 years old					
Number	3576	1500	314	572	−
Age (years)	54.1 (7.1)	55.5 (7.1)^a^	55.8 (7.8)^a^	56.9 (7.8)^ab^	<0.001
Body mass index (kg/m^2^)	22.0 (1.9)	27.0 (1.9)^a^	23.2 (1.4)^ab^	28.2 (2.6)^abc^	<0.001
Waist-to-height ratio	0.494 (0.044)	0.566 (0.042)^a^	0.520 (0.038)^ab^	0.589 (0.048)^abc^	<0.001
Mean arterial pressure (mmHg)	85.5 (12.7)	92.8 (13.5)^a^	92.5 (12.6)^a^	96.5 (13.2)^abc^	<0.001
Fasting blood glucose (mmol/L)	5.18 (0.82)	5.31 (0.77)^a^	7.06 (2.81)^ab^	6.45 (2.04)^abc^	<0.001
Total cholesterol (mmol/L)	5.33 (0.97)	5.44 (0.98)^a^	5.48 (1.07)^a^	5.45 (1.03)^a^	<0.001
Triglycerides (mmol/L)	1.16 (0.83)	1.39 (0.82)^a^	1.83 (1.37)^ab^	1.87 (1.29)^ab^	<0.001
LDL cholesterol (mmol/L)	3.33 (0.86)	3.50 (0.85)^a^	3.51 (0.99)^a^	3.51 (0.95)^a^	<0.001
HDL cholesterol (mmol/L)	1.46 (0.31)	1.36 (0.28)^a^	1.28 (0.28)^ab^	1.22 (0.25)^ab^	<0.001
Triglycerides/ HDL-C	0.90 (0.98)	1.11 (0.82)^a^	1.60 (1.54)^ab^	1.66 (1.43)^ab^	<0.001
hsCRP (μg/mL)	1.45 (3.31)	2.36 (4.07)^a^	2.24 (5.13)^a^	3.07 (4.18)^abc^	<0.001
Insulin (mIU/L)	5.30 (1.97)	6.69 (2.04)^a^	11.43 (3.34)^ab^	13.19 (3.94)^abc^	<0.001
HOMA-IR	1.22 (0.49)	1.56 (0.50)^a^	3.34 (0.91)^ab^	3.67 (1.30)^abc^	<0.001
eGFR (mL/min/1.73 m^2^)	96.8 (10.7)	95.3 (11.2)^a^	95.3 (12.5)	94.0 (12.6)^a^	<0.001
ACR (mg/g Cr)	11.5 (38.2)	17.5 (55.1)^a^	24.6 (83.9)^a^	34.9 (93.3)^abc^	<0.001
Renal function impairment, n (%)	187 (5.2)	145 (9.7)^a^	44 (14.0)^a^	125 (21.9)^abc^	<0.001

hsCRP, high sensitivity C-reactive protein; HOMA-IR, homeostasis model assessment-insulin resistance; MHNW, metabolic healthy norms-weight; MHO, metabolic healthy obesity; MUNW, metabolic unhealthy norms-weight; MUO, metabolic unhealthy obesity y; ACR, albumin-creatinine ratio; Cr, creatinine

^a,b,c^significant *posthoc* comparisons vs. MHNW, MHO, and MUNW, respectively

Data are presented as mean (*SD*) or percentage (%)

Among women under 45 years old, the metabolic biomarkers (i.e., TG and TG/HDL-C) were higher in the MUNW group than in the MHO group, without significant group differences in the ACR and prevalence of renal function impairment. Compared with the MUNW group, the MUO group had higher metabolic biomarkers (i.e., TG and TG/HDL-C) and prevalence of renal function impairment but had the same ACR.

Among women over 45 years old, the metabolic biomarkers (i.e., TG, HDL-C, and TG/HDL-C) were higher in the MUNW group than in the MHO group, but the ACR and prevalence of renal function impairment did not differ between these groups. The results of the comparison between the MUO and MUNW groups were different between men and women. In women, most of the metabolic biomarkers (i.e., TCHOL, TG, LDL-C, HDL-C, and TG/HDL-C) did not significantly differ between the MUO and MUNW groups, but the ACR and prevalence of renal function impairment were higher in the MUO group than in the MUNW group.

### Prevalence of metabolic syndrome components, according to the different metabolic body composition status

**Fig 1:**
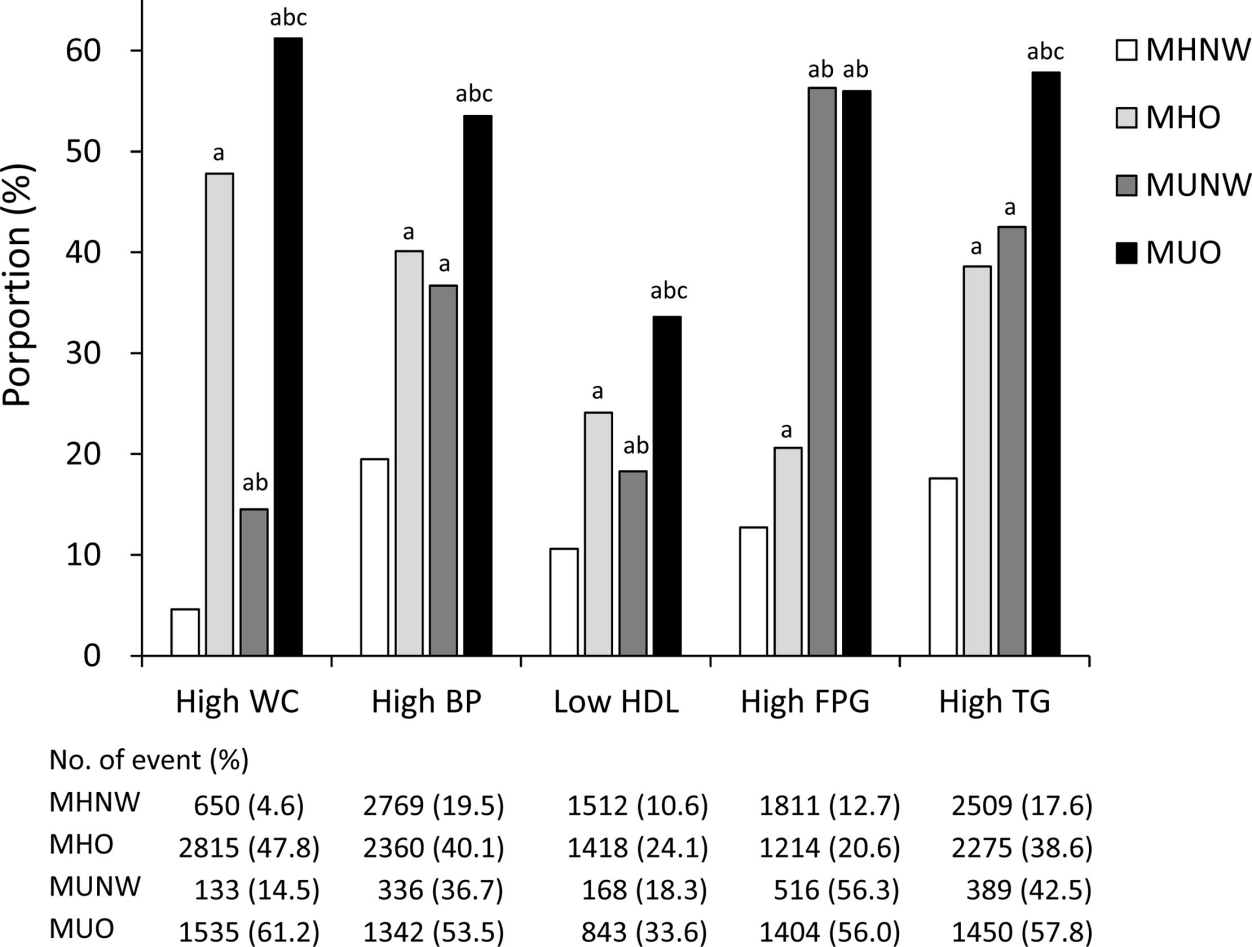
Prevalence of the metabolic components in the four study groups, according to the baseline metabolic health and obesity status. ^a,b,c^significant *posthoc* comparisons vs. MHNW, MHO, and MUNW, respectively MHO, metabolic healthy obesity; MUNW, metabolic unhealthy norms-weight; MUO, metabolic unhealthy obesity; FPG, fasting plasma glucose; TG, triglycerides.

The prevalence of high WC, BP, TG, and low HDL-C in the MUO group was significantly higher than those in the other groups. However, there was no significant difference in the prevalence of high FPG between the MUNW and MUO groups. Additionally, the FPG levels of the MUNW and MUO groups were higher, compared with those in the healthy and MHO groups, respectively. Moreover, there were no significant differences in the prevalence of high BP and TG between the MHO and MUNW groups (Fig 1).

### Association between metabolic body composition status and renal function impairment

Compared with the MHNW group, the MHO, MUNW, and MUO groups showed higher OR for renal function impairment in men of all ages (Table 4). However, that trend was no longer significant in men under 45 years old, after adjusting for the possible confounding factors. In contrast, in men over 45 years old, the risk for renal function impairment was greater in the MUNW group [OR 2.95, 95% confidence interval (CI) 2.02–4.30] and MUO group (OR 2.33, 95% CI 1.82–3.00), compared with that in the MHNW group. Similar results were observed in women. In women under 45 years old, the MHO, MUNW, and MUO groups demonstrated higher ORs (>1), compared with that in the MHNW group, but these effects reduced substantially and turned to be insignificant after adjustment. In women above 45 years old, after adjusting for confounding factors, the risk for renal function impairment was higher in the MUNW group (OR 1.95, 95% CI 1.35–2.82) and MUO group (OR 2.67, 95% CI 2.04–3.48), compared with that in the MHNW group.

**Table 4:** Combined effects of sex, age, and metabolic body composition status on renal function impairment.

			Odds ratio (95% confidence interval)	
Group / type	Number	Renal function impairment, n (%)	Unadjusted model	Adjusted model^a^
Men <45 years old				
MHNW	3131	68 (2.2)	1	1
MHO	1917	65 (3.4)	1.58 (1.12–2.23)*	0.80 (0.55–1.16)
MUNW	180	10 (5.6)	2.65 (1.34–5.24)*	1.58 (0.78–3.21)
MUO	876	81 (9.2)	4.59 (3.29–6.39)*	1.57 (1.08–2.30)*
Men ≥45 years old				
MHNW	3986	183 (4.6)	1	1
MHO	2103	150 (7.1)	1.60 (1.28–1.99)*	1.20 (0.96–1.52)
MUNW	272	40 (14.7)	3.58 (2.48–5.17)*	2.95 (2.02–4.30)*
MUO	908	141 (15.5)	3.82 (3.03–4.82)*	2.33 (1.82–3.00)*
Women <45 years old				
MHNW	3541	80 (2.3)	1	1
MHO	369	17 (4.6)	2.09 (1.22–3.57)*	1.44 (0.82–2.55)
MUNW	150	5 (3.3)	1.49 (0.60–3.74)	1.04 (0.40–2.70)
MUO	153	10 (6.5)	3.03 (1.54–5.96)*	1.43 (0.66–3.11)
Women ≥45 years old				
MHNW	3576	187 (5.2)	1	1
MHO	1500	145 (9.7)	1.94 (1.55–2.43)*	1.26 (0.99–1.60)
MUNW	314	44 (14.0)	2.95 (2.08–4.20)*	1.95 (1.35–2.82)*
MUO	572	125 (21.9)	5.07 (3.96–6.49)*	2.67 (2.04–3.48)*
Men and Women <45 years old#				
MHNW	6672	148 (2.2)	1	1
MHO	2286	82 (3.6)	1.64 (1.25–2.16)*	0.91 (0.66–1.23)
MUNW	330	15 (4.5)	2.10 (1.22–3.61)*	1.31 (0.75–2.29)
MUO	1029	91 (8.8)	4.28 (3.26–5.60)*	1.61 (1.16–2.23)*
Men and Women ≥45 years old#				
MHNW	7562	370 (4.9)	1	1
MHO	3603	295 (8.2)	1.73 (1.48–2.03)*	1.24 (1.05–1.46)*
MUNW	586	84 (14.3)	3.25 (2.52–4.19)*	2.38 (1.83–3.10)*
MUO	1480	266 (18.0)	4.26 (3.60–5.04)*	2.49 (2.08–2.99)*

MHNW, metabolic healthy norms-weight; MHO, metabolic healthy obesity; MUNW, metabolic unhealthy norms-weight; MUO, metabolic unhealthy obesity

^a^ Adjusted for mean arterial pressure, total cholesterol, triglycerides / HDL cholesterol, and hs-CRP level.

# Adjusted for sex, mean arterial pressure, total cholesterol, triglycerides / HDL cholesterol, and hs-CRP level

*P < 0.05.

In a supplemental analysis, we did not analyze separately by sex. In subjects under 45 years old, the MHO, MUNW, and MUO groups demonstrated significantly higher ORs (>1), whereas the effects substantially reduced after covariate adjustment. In subjects above 45 years old, the MHO, MUNW, and MUO groups demonstrated significantly higher ORs (>1) even after covariate adjustment (Table 4).

## Discussion

To our best knowledge, this was the first novel study that evaluated and compared the cardiovascular risks, microalbuminuria, and renal function impairment, according to sex, age, and different metabolic body composition status of a large Chinese population.

After adjusting for possible confounding factors, we found that among men under 45 years old, the MHO and MUNW groups had no significant differences in the metabolic biomarkers, ACR, and percentage of renal function impairment; whereas some metabolic biomarkers differed between the MUO and MUNW groups. For men over 45 years old, the metabolic biomarkers, ACR, and percentage of renal function impairment were higher in the MUNW group than in the MHO group. However, when the MUO and MUNW groups were compared, the metabolic biomarkers were significantly higher in the MUO group, but there were no differences in the eGFR, ACR, and percentage renal function impairment.

Unlike in men, women under 45 years old had higher metabolic biomarkers in the MUNW group than in the MHO group, although the ACR and percentage of renal function impairment did not significantly differ between these groups. A similar trend was found in women over 45 years old, unlike the findings in men. Moreover, in all ages, the metabolic biomarkers were significantly higher in the MUO group than in the MUNW group. Similar to the results in men, there were no differences in the eGFR, ACR between MUO and MUNW groups in women under 45 years old, but not in the percentage of renal function impairment. However, in women over 45 years old, the ACR and percentage of renal function impairment were significantly higher in the MUO group than in the MUNW group. The findings above implied that impaired renal function was independently associated with the status of metabolic obesity. However, this trend was observed only in elderly individuals (i.e., >45 years old), with significant sex difference.

A recent systematic review found that compared with healthy individuals, MHO individuals had higher rates of all-cause mortality and cardiovascular events and that there is no healthy pattern of increased weight [28]. Another large-scale study confirmed the trend of a higher prevalence of subclinical coronary atherosclerosis in MHO individuals than in metabolically healthy, normal-weight participants [29]; however, the difference became insignificant after adjusting for the metabolic risk factors and LDL-C. On the other hand, a population-based prospective cohort study on 61,299 individuals suggested that compared with metabolically healthy normal-weight subjects, MHO individuals were not at increased risk for acute myocardial infarction (AMI) [30]. One possible mechanism for the lower risk for AMI in MHO individuals was the reduced visceral fat mass and the markedly reduced liver fat content. Variability in the adiponectin receptor 1 gene, which correlates with lower levels of the liver-secreted glycoprotein fetuin-A, might determine the prevalence of MHO. Above mechanisms affect the metabolism of glucose and lipids and induces subclinical inflammation [31]. A concept introduced by Ruderman et al [32], MUNW individuals were characterized by metabolic complications that were similar to those in obese individuals. The major factor for the difference in the cardiovascular risk between MUNW and MHO individuals was the extent of visceral fat accumulation [33]. A report from the Third National Health and Nutrition Examination Survey (NHANES III) in the Unites States concluded that normal-weight obesity (NWO), which was defined as MUNW in this present study, was associated with a relatively high cardiovascular risk [34]. The trend was independently significant, especially in women.

A recent cohort study confirmed the association of MHO with an increased incidence of CKD [35]. Another prospective study found that the risk for CKD was the highest in MUO [hazard ratio (HR) 1.56], followed by MHO (HR 1.38) and MUNO (HR 1.37), compared with the CKD risk in healthy individuals [36]. Although the mechanism of chronic obesity contributing to CKD remains elusive, multiple mechanisms had been proposed and include glomerular hyperfiltration, development of microalbuminuria/ proteinuria, increased glomerular capillary wall tension, and podocyte stress; these are followed by hypofiltration, decreased eGFR, and CKD progression [37]. Adipose tissue-derived factors, such as tumor necrosis factor-α, interleukin-6, and plasminogen activator inhibitor-1, might contribute to renal function impairment. Furthermore, adiposity can lead to ectopic lipid accumulation in the kidneys and cause structural and functional changes that mediate certain renal diseases [38]. In addition to adiposity itself, high caloric diet leading to obesity may increase the CKD risk, through the circuitous loop among Sirt1, adiponectin, and podocyte effacement [39]. The impact of obesity on the pathogenesis of CKD appeared to be independent of blood pressure and the presence of diabetes mellitus, which are the two most common causes of CKD [40]. On the other hand, another recent study, which followed-up 3,136 subjects for eight years and divided the subjects in a similar way as our current study, found higher risks for CKD and proteinuria in MUO but not in MHO [41]. In this study, after dividing the study population into different sex and age groups, we found that impaired renal function was independently associated with the status of metabolic obesity. However, the trend was observed only in the elderly (over 45 years old), with significant sex difference.

A study that enrolled a Japanese population found a sex difference in the association between BMI and impaired renal function and suggested that increased BMI was a risk factor for impaired renal function in men but not in women [42]. A similar finding was previously observed in another cohort, suggesting that the sex difference might be partly attributable to the prevalence of cigarette smoking [43]. There had been studies that addressed sex differences in the progression of certain renal diseases. Compared with women, men were found to have a higher risk for CKD and ESRD, even at a young age [44]. An animal study suggested that the sex differences may partly be caused by the influence of testosterone and the other sex hormones on the risk for proteinuria and glomerular sclerosis [45]. In the current study, individuals over 45 years old had higher ACR and percentage of renal function impairment in the MUNW group than in the MHO group; this trend was obvious in men but not in women. When renal function was compared between the MUO and MUNW groups, significantly higher ACR and percentage of renal function impairment were in women but not in men. A further study would be needed to clarify the mechanism for these differences between sexes.

Since we divided the participants into four groups of metabolic body composition according to HOMA-IR and BMI, we also observed that HOMA-IR was significantly different between four groups in all ages and both genders. The highest HOMA-IR value was found in MUO group, followed by MUNW, MHO and MHNW. The finding was compatible with previous studies, with the finding of progressive increase in HOMA-IR values with increasing BMI [26, 46]. As for the level of hs-CRP, from previous studies, high hs-CRP levels were suggested to be predictive of CKD for women but not for men in an 11-year prospective cohort study [47]. While another 15-year follow-up study in the Beaver Dam Chronic Kidney Disease Study found that hs-CRP levels were not associated with the risk of CKD [48]. In the current study, higher hs-CRP level was observed in obesity groups include MHO and MUO comparing to MHNH among both genders younger than 45 years old and among women older than 45 years old. While significant higher hs-CRP level was only observed in MUO comparing to MHNH and MHO groups among men older than 45 years old. The result that higher hs-CRP observed in obesity groups was compatible with our previous study [49]. Further study and analysis would be needed to evaluate the relationship of hs-CRP and renal impairment.

The strength of the current study was the relative large sample size, which enabled division into different groups of sex and age and provided relatively convincing results. However, there remained some limitations in this study. First, the cross-sectional study design precluded the establishment of a casual or pathophysiologic relationship between the anthropometric measurements and the factors. Second, ACR was measured only once and not within a period of three months or longer; this could have resulted in misleading classifications of albuminuria and improper exclusion of cases with acute renal injury. Third, we did not evaluate some of the uncertainties associated with proteinuria, such as current use of certain antihypertensive medicines, diet, and physical activity. Fourth, insulin resistance was defined by HOM-IR and not by the invasive and time-consuming euglycemic insulin clamp. Fifth was the possible selection bias, because the study subjects were recruited from a health checkup program in a local hospital. Fifth, due to the limitation of not checking estrogen during health checkup, the results might not be able to generalize to normal population without excluding the effect of female hormone, we selected the cut-off value of 45 years old based on our previous studies and references [49, 50]. Sixth, based on the setting of retrospective cross-sectional study, we did not include the data of intensity and regularity of exercise, which would be of great value evaluating related risks.

## Supporting information

S1 File Data set. (XLSX)
